# A comparison of self-reported COVID-19 symptoms between android and iOS CoronaCheck app users

**DOI:** 10.1038/s41746-025-01595-1

**Published:** 2025-04-09

**Authors:** Michael Winter, Thomas Probst, Thomas Keil, Rüdiger Pryss

**Affiliations:** 1 Institute of Clinical Epidemiology and Biometry, University of Würzburg, Würzburg, Germany; 2 Institute of Medical Data Science, University Hospital of Würzburg, Würzburg, Germany; 3 Division of Psychotherapy, Department of Psychology, Paris Lodron University Salzburg, Salzburg, Austria; 4 State Institute of Health I, Bavarian Health and Food Safety Authority, Erlangen, Germany; 5 Institute of Social Medicine, Epidemiology and Health Economics, Charité—Universitätsmedizin Berlin, Berlin, Germany

**Keywords:** Computational biology and bioinformatics, Health care, Medical research, Signs and symptoms

## Abstract

This study explored differences in COVID-19 infections and symptoms between Android and iOS users using data from the CoronaCheck app. This cross-sectional analysis included 23,063 global users (20,753 Android and 2310 iOS) from April 2020 to February 2023. Participants reported COVID-19 symptoms and contact risks, with data analyzed to adjust for age, sex, education, and country. Android users were generally younger, more often male, had a lower educational level, and reported more symptoms on average (2.1 vs. 1.6) than iOS users. Android users also had higher suspected COVID-19 infection rates (24% vs. 11%), with an adjusted odds ratio of 2.21 (95% CI: 1.93–2.54). These findings suggest platform-based differences in COVID-19 infection rates and symptom reporting, highlighting potential biases in mobile health research. Adjusting for device operating systems may be crucial in improving the reliability of population-based health data collected through mobile platforms.

## Introduction

Mobile health (mHealth), referring to the integration of smartphone technology into clinical and population-based research, is rapidly developing^1,2^. However, mHealth studies encounter challenges, including underutilization of the extensive capabilities of smartphones (e.g., sensor-based data collection), diminished participant motivation, and poor adherence^3,4^. These limitations may lead to systematic differences in data quality and engagement across user groups, influencing the generalizability and reliability of digital health assessments.

The relevance of distinguishing between mobile operating systems (OS) was emphasized, for instance, in the TrackYourTinnitus app study, where Android users were older and reported a longer disease duration than iOS users^5–7^. Another study from TrackYourHearing revealed that iOS users experienced more positive emotional states than Android users, reporting lower levels of irritability and exhaustion^8^. These findings suggest that differences in OS usage may reflect underlying demographic, behavioral, or psychological factors that influence health-related self-assessments. Moreover, this highlights the crucial role of technological factors in shaping emotional experiences among individuals with hearing loss, reinforcing the need to account for platform-based biases in mHealth research. As mHealth technology continues to evolve, understanding these distinctions is essential for optimizing app design, improving data quality, and enhancing user engagement^6^.

Few studies have directly investigated health-related differences between Android and iOS users, despite growing evidence of behavioral and sociodemographic distinctions between these groups^6,9,10^. For example, in the context of anti-smoking interventions, iOS users were more likely to download smoking cessation apps with the intention of making a quit attempt, had a history of more recent quit attempts, and were less likely to use pharmacological aids such as nicotine replacement therapy or alternative medication compared to Android users^11^. These differences suggest that motivations for health-related app usage may be shaped not only by individual behavioral patterns but also by economic and social factors, such as income levels, brand perception, and marketing strategies associated with different operating systems. However, other research has found no significant association between smartphone OS and daily health habits^12^, highlighting the complexity of disentangling technological and personal influences on health behaviors. Given the platform-based differences observed in mobile health data collection, further research is necessary to establish a clearer understanding of existing mechanisms on how user demographics and behavioral tendencies interact with OS-based variations in digital health engagement^13^. Such insights could provide a stronger foundation for optimizing app-based health interventions and ensuring equitable digital health access across different user populations.

Despite the growing integration of mobile technology in health surveillance, population-based studies that directly compare users of different mobile operating systems in relation to COVID-19 infections and symptom reporting remain limited^1^. Research exploring OS-based differences in COVID-19 data has largely focused on variations in app availability and functionality across Google’s and Apple’s app stores^14,15^, overlooking potential behavioral or reporting biases linked to the technology itself. Understanding these distinctions is crucial, as the decision to use a specific OS may be influenced by multiple factors—economic status, social preferences, digital literacy, or perceived brand prestige—that could also affect users’ engagement with health monitoring tools. To address this gap, our study examined the association of suspected COVID-19 infections based on self-reported symptoms and recent contact with confirmed COVID-19 cases and sociodemographic characteristics among Android and iOS users of the *CoronaCheck* app from April 2020 to February 2023. By establishing a foundation for further research in this domain, our findings contribute to a more nuanced understanding of how technological and social factors intersect in shaping digital health behaviors and outcomes.

During the COVID-19 pandemic, numerous mobile applications were developed to monitor symptoms and assist in public health efforts^16–19^. One of the first of its kind was the non-commercial *CoronaCheck* app, developed through a collaboration between German medical universities and the Bavarian public health authority. Launched in early 2020, its primary goal was to alleviate the burden on overwhelmed telephone hotlines and provide immediate, reliable self-assessment for suspected COVID-19 cases^19^. Additionally, its design was tailored to maximize accessibility and reach a large and diverse population by ensuring ease of use, broad device compatibility, and compliance with data protection regulations, thereby facilitating widespread adoption and participation in digital health surveillance efforts. The app guided users through a structured questionnaire based on official recommendations from the Robert Koch Institute (RKI), assessing symptoms and recent exposure history in real-time. Importantly, *CoronaCheck* functioned not only as a digital triage tool but also as a behavioral intervention by influencing users’ risk perceptions and subsequent health-seeking actions^18^. By integrating patient-reported outcomes with a standardized symptom assessment framework, the app provided tailored recommendations, potentially impacting users’ likelihood of seeking testing or medical consultation. Moreover, its adherence to European data protection laws and the Medical Device Regulation (MDR)^16,17,20^ ensured compliance with ethical and privacy standards while enabling valuable epidemiological data collection.

## Results

### Sociodemographic characteristics of participants

In general, the evaluation included assessments from a total of 23,063 users, comprising 20,753 Android and 2310 iOS. Nearly sixty percent of the participating *CoronaCheck* users who gave permission to collect GPS data came from only three countries, Germany (31.9%; 7358 users), India (13.9%: 3222) and South Africa (12.9%; 2978). A further four percent were from The Netherlands (2.1%; 477) and Bangladesh (1.5%; 358). An additional 10.3% of users represented various countries worldwide. The remaining 27.3% of users did not provide any information regarding their locality.

The study included approximately 90% Android and 10% iOS users, which aligns with the worldwide mobile OS market share^21^. However, a comparison with country-specific distributions suggests that our sample does not fully represent the distribution of Android and iOS users in some regions (e.g., underrepresentation of iOS in India; see Supplementary Table 1 for a detailed breakdown of Android and iOS users).

Android users, as compared to iOS, were considerably younger (62.5% (Android) vs 41.5% (iOS) under 40 years) and slightly more often male (64% (Android) vs 60.3% (iOS)). They also seemed to have spent fewer years in school than iOS users (32.8% (Android) vs 23.8% (iOS), although a considerable number of participants did not answer this question (27.3% (Android) vs 15.4% (iOS); see Table 1).

**Table 1:** Sociodemographic characteristics of Android versus iOS users of the *CoronaCheck* self-help app

Characteristic	Android (*N* = 20,753)	iOS (*N* = 2310)	*p*-Value
*Age (years)*			
20–29	36.6%	21.9%	
30–39	25.9%	19.7%	
40–49	14.4%	18.0%	
50–59	10.3%	17.7%	<0.01
60–69	6.7%	13.5%	
70–79	4.1%	7.6%	
80+	2.0%	1.6%	
*Sex*			
Female	35.2%	38.8%	
Male	64.0%	60.3%	<0.01
Diverse or not reported	0.8%	0.9%	
*Education*			
<12 years	32.8%	23.8%	
12+ years	39.9%	60.8%	<0.01
Not reported	27.3%	15.4%	
*Country*			
Germany	31.4%	36.6%	
India	15.4%	0.7%	
South Africa	13.7%	6.2%	
Netherlands	0.5%	16.7%	<0.01
Bangladesh	1.6%	1.3%	
Others	9.3%	18.7%	
Not reported	28.1%	19.8%	

This table presents the sociodemographic characteristics of Android and iOS users of the *CoronaCheck* app, highlighting significant differences in age distribution, sex, education level, and country of residence. Android users were generally younger, with 36.6% aged 20–29 years compared to 21.9% of iOS users, while older age groups (50+ years) were more prevalent among iOS users (40.4% vs. 23.1% in Android users). Regarding sex distribution, a higher proportion of Android users were male (64.0% vs. 60.3% in iOS users), whereas females were slightly more prevalent among iOS users (38.8% vs. 35.2%, *p* < 0.01). Educational attainment also differed between platforms, with a greater share of iOS users having 12 or more years of formal education (60.8% vs. 39.9%), while Android users more frequently had less than 12 years of education (32.8% vs. 23.8%, *p* < 0.01). The distribution by country varied significantly, with Germany having a higher proportion of iOS users (36.6% vs. 31.4%), whereas India (15.4% vs. 0.7%) and South Africa (13.7% vs. 6.2%) had significantly more Android users. Additionally, 16.7% of iOS users were from the Netherlands compared to only 0.5% of Android users (*p* < 0.01). These statistically significant differences (*p* < 0.01) highlight important demographic variations between Android and iOS users, providing crucial context for interpreting platform-based differences in reported COVID-19 symptoms and infection risk.

*Note:* Statistically significant differences (*p*-value < 0.05) suggest potential differences in sociodemographic distributions for age, sex, education, and country between Android and iOS users.

### COVID-19 related symptoms

On average, Android users reported more COVID-19-associated symptoms than iOS users (2.13 (Android) vs 1.62 (iOS)). The two most common reported symptoms in both user groups were headache (29.6% (Android), 24.8% (iOS)) and cough (27.3% (Android), 22.5% (iOS)). Most COVID-19 symptoms, except runny nose and sore throat, differed between iOS and Android users. Android users had more often headache (29.6%), cough (27.3%), weakness (26.2%), muscle pain (24.4%), fever (21.6%), breathlessness (16.1%), loss of smell (12.8%), loss of taste (12.5%), but iOS users had more often diarrhea (2.7%; see Table 2).

**Table 2:** Self-reported potential COVID-19 symptoms of Android versus iOS users of the *CoronaCheck* self-help app

Symptoms	Android (*N* = 20,753)	iOS (*N* = 2310)	*p*-Value
Number of symptoms [mean (SD)]	2.13 (2.67)	1.62 (2.13)	<0.01
Headache [%]	29.6	24.8	<0.01
Cough [%]	27.3	22.5	<0.01
General weakness [%]	26.2	17.1	<0.01
Muscle Pain [%]	24.4	18.5	<0.01
Fever [%]	21.6	11.0	<0.01
Runny Nose [%]	21.5	21.4	0.97
Sore Throat [%]	20.0	20.2	0.85
Breathlessness [%]	16.1	12.1	<0.01
Loss of Smell [%]	12.8	6.1	<0.01
Loss of Taste [%]	12.5	6.0	<0.01
Diarrhea [%]	0.7	2.7	<0.01

The table details the self-reported COVID-19-related symptoms among Android and iOS users of the *CoronaCheck* app, highlighting differences in the number and type of symptoms reported. On average, Android users reported a higher number of symptoms per person (mean = 2.13, SD = 2.67) compared to iOS users (mean = 1.62, SD = 2.13, *p* < 0.01). Several symptoms were significantly more common among Android users, including headache (29.6% vs. 24.8%), cough (27.3% vs. 22.5%), general weakness (26.2% vs. 17.1%), muscle pain (24.4% vs. 18.5%), fever (21.6% vs. 11.0%), breathlessness (16.1% vs. 12.1%), loss of smell (12.8% vs. 6.1%), and loss of taste (12.5% vs. 6.0%) (all *p* < 0.01). In contrast, diarrhea was more frequently reported by iOS users (2.7% vs. 0.7%, *p* < 0.01). The statistically significant variations (*p* < 0.01) suggest notable differences in symptom reporting between Android and iOS users, potentially reflecting differences in user demographics, health status, or reporting behaviors.

*Note:* Statistically significant differences (*p*-value < 0.05) suggest potential variations in the number and distribution of symptoms between Android and iOS users.

### COVID-19 infection status

Android users, compared to iOS users, were twice as often classified as having a suspected COVID-19 infection based on reported symptoms (24.0% (Android) vs 11.1% (iOS)) or, as asymptomatic but with high risk due to a recent contact to someone with a confirmed COVID-19 infection (4.1% (Android) vs 2.4% (iOS); see Table 3). Information on symptoms unrelated to COVID-19 was similar on both platforms (43.1% (Android) vs 46.0% (iOS). Finally, iOS users more frequently reported having no COVID-19-related symptoms and no history of suspected COVID-19 contact (28.8% (Android) vs 39.6% (iOS)).

**Table 3:** COVID-19 status by the *CoronaCheck* app based on self-reported symptoms and contacts, stratified for Android and iOS users

Final assessment based on self-reported symptoms and potential contacts	Android (*N* = 20,753)	iOS (*N* = 2310)	*p*-Value
A) Suspected COVID-19 infection	24.0%	11.1%	
B) Current symptoms without suspicion of COVID-19	43.1%	46.9%	
C) Currently no symptoms, but at risk due to recent contact with a confirmed COVID-19 case	4.1%	2.4%	<0.01
D) No symptoms, no recent contact	28.8%	39.6%	

This table summarizes the COVID-19 status classifications based on self-reported symptoms and recent contact with confirmed cases among Android and iOS users of the *CoronaCheck* app. The table presents four possible COVID-19 status outcomes: suspected COVID-19 infection, symptoms without suspicion of COVID-19, recent contact with a confirmed case without symptoms, and no symptoms or recent contacts. Android users were more frequently classified as having a suspected COVID-19 infection (24.0% vs. 11.1%), while iOS users were more often categorized as having no symptoms and no recent contact (39.6% vs. 28.8%). Additionally, Android users had a slightly higher proportion of individuals at risk due to recent contact with a confirmed case (4.1% vs. 2.4%). These statistically significant differences (*p* < 0.01) highlight notable platform-based variations in self-reported COVID-19 status, potentially influenced by differences in symptom perception, reporting behavior, or exposure risk.

*Note:* Statistically significant differences (*p*-value < 0.05) suggest potential differences in assessments of suspected COVID-19 infection between Android and iOS users.

### Association of user platforms with suspected infection and COVID-19 symptoms

After adjusting for the reported sociodemographic variables, Android users were 2.2 times more likely to be classified as having a suspected COVID-19 infection than iOS users (AOR = 2.21; 95% CI: 1.93–2.54). The likelihood of having a suspected COVID-19 infection was lower for women (AOR = 0.75; 95% CI: 0.70–0.80), younger users (AOR = 0.78; 95% CI: 0.76–0.80), and those with less than 12 years of schooling (AOR = 1.23 for years >12; 95% CI: 1.08–1.29). These associations remained relatively stable after mutual adjustments (see Table 4).

**Table 4:** The association between user status (Android vs. iOS) and the outcomes “Suspected COVID-19 infection”, “Loss of smell”, and “Loss of taste”

	Crude Odds Ratio [95% CI]	p-value	Adjusted Odds Ratio [95% CI]	p-value
*Outcome “Suspected COVID-19 infection*” *(vs not)* *	*N* *=* *23,063*			
Android user (ref. iOS user)	2.53 [2.21–2.89]	<0.01	2.21 [1.93–2.54]	<0.01.
Female sex (ref. male)	0.76 [0.72–0.81]	<0.01.	0.75 [0.70–0.80]	<0.01
Diverse and not reported sex (ref. male)	2.98 [2.26–3.83]	<0.01	3.87 [2.86–5.24]	<0.01
Age (per 10y-category)	0.72 [0.71–0.74]	<0.01	0.78 [0.76–0.80]	<0.01
Schooling 12+ y (ref. < 12 y)	1.35 [1.30–1.41]	<0.01	1.23 [1.08–1.29]	<0.01
Not reported (ref. < 12y)	1.81 [1.68–1.96]	<0.01	1.48 [1.36–1.61]	<0.01
Country (ref. Germany)				
The Netherlands	0.84 [0.56–1.26]	0.39	0.80 [0.51–1.26]	0.34
India	11.77 [10.48–13.21]	<0.01	9.64 [8.36–11.12]	<0.01
Bangladesh	17.97 [14.31–22.58]	<0.01	13.22 [10.27–17.02]	<0.01
South Africa	2.59 [2.26–2.97]	<0.01	1.81 [1.54–2.11]	<0.01
Others	7.70 [6.79–8.73]	<0.01	5.96 [5.18–6.86]	<0.01
Not reported	5.95 [5.34–6.63]	<0.01	4.52 [4.02–5.08]	<0.01
*Outcome “Loss of smell" (vs no)* *	*N* *=* *23,063*			
Android user (ref. iOS user)	2.26 [1.89–2.69]	<0.01	1.99 [1.67–2.39]	<0.01
Female sex (ref. male)	0.83 [0.76–0.90]	<0.01	0.82 [0.76–0.89]	<0.01
Diverse and not reported sex (ref. male)	2.56 [1.87–3.50]	<0.01	2.91 [2.11–4.02]	<0.01
Age (per 10 y-category)	0.77 [0.74–0.79]	<0.01	0.81 [0.79–0.84]	<0.01
Schooling 12+ y (ref. < 12y)	1.21 [1.15–1.27]	<0.01	1.11 [1.06–1.17]	<0.01
Not reported (ref. < 12y)	1.46 [1.32–1.62]	<0.01	1.23 [1.11–1.37]	<0.01
Country (ref. Germany)				
The Netherlands	0.98 [0.63–1.54]	0.94	1.10 [0.66–1.84]	0.70
India	5.54 [4.81–6.37]	<0.01	4.73 [3.98–5.63]	<0.01
Bangladesh	6.55 [5.01–8.57]	<0.01	5.00 [3.70–6.74]	<0.01
South Africa	2.64 [2.25–3.09]	<0.01	1.91 [1.59–2.30]	<0.01
Others	4.89 [4.21–5.69]	<0.01	3.79 [3.19–4.49]	<0.01
Not reported	3.70 [3.25–4.22]	<0.01	3.02 [2.62–3.49]	<0.01
Outcome “Loss of taste" (vs no) *	*N = 23,063*			
Android user (ref. iOS user)	2.22 [1.89–2.69]	<0.01	2.00 [1.67–2.39]	<0.01
Female sex (ref. male)	0.93 [0.85–1.00]	0.06	0.92 [0.85–1.00]	0.06
Diverse and not reported sex (ref. male)	2.81 [2.06–3.83]	<0.01	3.17 [2.30–4.38]	<0.01
Age (per 10 y-category)	0.78 [0.76–0.81]	<0.01	0.83 [0.79–0.84]	<0.01
Schooling 12+ y (ref. <12 y)	1.23 [1.16–1.29]	<0.01	1.14 [1.08–1.20]	<0.01
Not reported (ref. <12 y)	1.50 [1.35–1.66]	<0.01	1.28 [1.15–1.42]	<0.01
Country (ref. Germany)				
The Netherlands	0.75 [0.45–1.26]	0.28	0.67 [0.38–1.17]	0.16
India	5.56 [4.83–6.40]	<0.01	5.20 [4.36–6.20]	<0.01
Bangladesh	6.76 [5.17–8.83]	<0.01	5.61 [4.15–7.59]	<0.01
South Africa	2.53 [2.15–2.98]	<0.01	1.97 [1.63–2.38]	<0.01
Others	4.79 [4.11–5.58]	<0.01	3.83 [3.22–4.54]	<0.01
Not reported	3.63 [3.25–4.15]	<0.01	3.03 [2.62–3.49]	<0.01

The table presents the association between user platform (Android vs. iOS) and COVID-19-related outcomes, including suspected COVID-19 infection, loss of smell, and loss of taste. The table provides both crude and adjusted odds ratios (ORs) with 95% confidence intervals for each outcome, adjusted for sociodemographic variables such as sex, age, education, and country of residence. Results show that Android users had higher odds of suspected COVID-19 infection, loss of smell, and loss of taste compared to iOS users, even after adjusting for sociodemographic factors. Other sociodemographic factors show significant variations in suspected COVID-19 infection, loss of smell and taste. The associations are statistically significant (*p* < 0.05), highlighting platform-based differences in symptom reporting and COVID-19 risk perception among app users.

*Note:* The association between user status was analyzed in three separate multiple regression models, each adjusted for sex, age, education level, and country (crude, adjusted odds, and 95% CI). Statistically significant differences (*p*-value < 0.05) suggest potential differences in sociodemographic characteristics between Android and iOS users. *Based on self-reported COVID-19 symptoms and recent contact with a confirmed COVID-19 case.

Regarding the country of stay during the use of the app, participants from the Netherlands had a similar likelihood of a suspected COVID-19 infection than those from neighboring Germany (*p*-value = 0.34). Participants from all other countries, especially from South Asia, were much more likely to be classified as having a COVID-19 infection than those from Germany and The Netherlands (AOR between 9.64; 95% CI: 8.36–11.12 for India and 13.22; 95% CI: 10.27–17.02 for Bangladesh). These associations seemed relatively stable after adjusting for sociodemographics (see Table 4).

The results for the two outcomes loss of smell and loss of taste were similar as for the outcome suspected COVID-19 infection. Although the associations were in the same direction, the likelihoods for Android vs iOS users and for most sociodemographic variables were slightly lower (see Table 4).

Detailed comparisons of absolute differences and attributable proportions for suspected COVID-19 infection, loss of smell, and loss of taste are available as additional tables (see Supplementary Tables 2–4) in the Supplementary Material.

## Discussion

The *CoronaCheck* app was mostly used in three countries (Germany, India, and South Africa). Android users were younger, slightly more often male, and had a lower educational level in terms of years in school than iOS users. Android users were more likely to have a suspected COVID-19 infection than iOS users. Furthermore, they reported more COVID-19-associated symptoms on average. Adjusting for sociodemographic factors reduced these associations slightly. Female sex, younger age, and a lower educational level were associated with a suspected COVID-19 infection. Users from Germany and The Netherlands were considerably less likely to have a suspected COVID-19 infection than users from other regions.

In comparison with other studies, sex-/gender differences play a significant role in COVID-19 outcomes, including symptom reporting and health experiences. A worldwide systematic review from 2020 found that both biological (i.e., sex-related) and sociocultural (i.e., gender-related) factors contribute to variations in how men and women experience and report COVID-19 symptoms^22^. The review confirmed our observation that men had a higher proportion of suspected COVID-19 infections and were more likely to present with severe outcomes compared to women. A study from 2024 in the U.S. with 23,824 participants showed that males were more likely to report loss of smell and taste and speculated that this may be due to a more robust response to infections of female immune systems^23^.

In the context of age, our results showed that younger individuals were more likely to report symptoms and to be associated with COVID-19 infection compared to older persons. Interestingly, participants in the over-80 age group showed the opposite effects, with higher COVID-19 infections and the prevalence of loss of smell and taste symptoms. This was shown in a large-scale study from 2020 with over 2.6 million participants from the US and UK^16^. A comprehensive analysis conducted in European countries of self-reported COVID-19 symptoms from 2021 observed a higher prevalence of symptoms such as loss of smell and taste among younger individuals, consistent with our findings of age-related differences in symptom reporting^24^. Juxtaposed to our findings, analyses on the TrackYourTinnitus app from 2018 and 2023 with 1517 and 2693 users, respectively, observed that Android users tended to be older on average^6,7^.

This study did not include children, and therefore, the findings are not generalizable to this age group. While older adults were part of the sample, we did not assess potential differences in accessibility between Android and iOS users within this demographic. Variations in digital literacy, device usability, and app engagement may influence how older adults interact with mHealth applications, potentially affecting symptom-reporting behavior. Prior research suggests that older adults often encounter challenges in using mHealth applications due to digital literacy gaps, cognitive limitations, and motor skill constraints^25^. However, other studies indicate that users across the full age spectrum can effectively utilize the basic functionalities of mHealth apps, as demonstrated in a usability study of the Dutch COVID-19 contact tracing app^26^.

Higher education levels were correlated with an increased likelihood of reporting both smell and taste loss, as well as being classified with suspected COVID-19 infection, suggesting greater health consciousness or an enhanced ability to articulate and report symptoms among more formally educated persons. Our findings confirm comprehensive reviews, highlighting education as a key factor in health-reporting behavior and symptom awareness in general. Studies showed higher education levels correlate with better health reporting and awareness of symptoms^27,28^.

App users from India, Bangladesh, and South Africa were more likely to report loss of smell, loss of taste, and suspected COVID-19 infection than those from Germany. This was in line with previously reported higher prevalence estimates of loss of smell and taste during COVID-19 infections in India and Bangladesh^29^. A meta-analysis on COVID-19 symptoms from 2021 considering different countries found that cultural factors (e.g., cultural norms leading to underreporting of sensory symptoms) and public health reporting standards (e.g., guidelines prioritizing other symptoms) also affect how COVID-19 symptoms like smell and taste loss were documented, with evidence suggesting that Western countries like Germany report such symptoms less frequently^30^.

The results indicate that users from India and Bangladesh had significantly higher odds of suspected COVID-19 infection and symptoms like loss of small and taste. In regions such as India and Bangladesh, official COVID-19 prevalence estimates were often hampered by limited testing capacity, especially in the early phases of the pandemic^31^. This likely led to an underestimation of the true infection burden in these populations, making direct comparisons to official case numbers challenging. The increased odds observed in our data may, in part, reflect a more accurate picture of the underlying disease burden, particularly among younger individuals, who represented a larger proportion of our sample in these regions^32^. Additionally, the younger demographic in these countries may have contributed to a higher likelihood of symptom reporting through our app, as younger individuals tend to be more engaged with digital health platforms.

Previous studies during the pandemic have shown that country-specific factors influence COVID-19 symptom reporting and app usage. For example, health behaviors such as trust in health authorities and perceived personal risk of infection impact how individuals engage with COVID-19 apps^18,33,34^. Countries with widespread infections and advanced digital infrastructure tend to see higher app adoption and more frequent symptom reporting (e.g., UK and US). The results align with our findings that health apps, especially those with consultation features, were more likely to be adopted. Country-specific differences related to COVID-19 incidences, such as those observed in our analysis from users in India, Bangladesh, and South Africa, suggest that cultural context and local health communication strategies also play a role in how symptoms are reported, as shown by an international study from 2020 with 29 countries, including the 5 predominant countries of app users in our study^35^.

Comparisons of Android with iOS users showed that the latter more often have a higher socioeconomic status, reflected in higher educational and income levels, as demonstrated in a worldwide 3-year long-term study with 1608 subjects^36^. Android users were often younger and represented a more diverse range of socioeconomic backgrounds, reflecting broader accessibility and affordability across different income levels^37^. A study on smoking cessation apps from 2017 with 1368 users found that iOS users were more likely to engage consistently in health-conscious behaviors, including the use of app tools to quit smoking and tracking progress^11^.

Differences in symptom reporting behavior between Android and iOS users may also be influenced by platform-specific technical characteristics^38^. Previous studies have shown that iOS enforces stricter privacy controls and background app restrictions, which can limit continuous data collection compared to Android, potentially leading to underreporting^39^. Additionally, differences in app design and user engagement patterns may contribute to variations in reporting behavior, with Android apps often emphasizing active user input, while iOS apps focus more on data visualization^40^. The accessibility of Android devices across a broader range of socioeconomic backgrounds may further increase sample diversity^41^. While personality differences between iOS and Android users have been suggested, a large-scale study found that these differences were negligible after accounting for sociodemographic factors^42^.

Although controlling for educational level explained some of the association between users with different mobile operating systems and COVID-19 infection in our study, most of the underlying mechanisms of the differences remain unclear. Since we used only educational level as a proxy for social status, other socioeconomic parameters that we did not examine, like household income or employment status may play a role. It has been shown that living in urban versus rural environments effects smartphone usage of Android users, thus future studies should include this information as a potential confounder^43^. Personality trait differences between iOS and Android users have been suggested, however, in an international study and a study from Germany, both from before the COVID-19 pandemic, no considerable personality differences were found between the two user groups after sociodemographics had been controlled for^44^.

Regarding public health implications, the non-commercial *CoronaCheck* app was developed for individuals seeking free and instant advice about a possible COVID-19 infection as well as supporting public health authorities’ efforts in the containment of the COVID-19 virus spread. Our app was able to offer many of the functionalities that a telephone hotline of authorities (staffed by many employees) or health insurance companies offer. Thus, it may not fully replace but certainly complement them. The present study did not examine directly if these general aims were achieved, but its findings will help tailor digital health tools for public health interventions considering specific target groups’ technological preferences, age, sex, educational level, and regional context. In addition, such tools can be easily used for accompanying evaluation research, as our project showed, where most users were willing to participate and to enter sociodemographic and medical information for (anonymous) research. If promoted well and used widely during a pandemic, the app’s geolocation information (here, over 70% of the users agreed) may assist in monitoring viral spread across regions.

Our study had several strengths, including the large sample size of over 23,000 users of one of the first freely available self-help app for mobile phones during the COVID-19 pandemic, which adhered to the medical device regulations and was available in both Google’s Play and Apple’s App Store. The app’s global usage provided diverse demographic data, offering insights into different population segments across countries. Additionally, the app assessed a wide range of COVID-19-associated symptoms using a standardized questionnaire that was kept short to achieve a high response quote.

However, there are also limitations that should be noted. First, the voluntary participation in the study may have introduced self-selection bias. While the proportions of Android (≈90%) and iOS (≈10%) users in our study align with global OS distributions, and the included sociodemographic characteristics reflect real-world trends in many regions, our sample is not necessarily representative of all Android and iOS users. Nearly 60% of participants came from only three countries (Germany, India, and South Africa), which may limit the generalizability of our findings. Furthermore, as the *CoronaCheck* app was only available in German and English, this may have resulted in a linguistically homogeneous user base, potentially influencing symptom reporting patterns.

Second, our findings should not be interpreted as representative of the countries included, as participants were not selected through random sampling, and the dataset includes only limited sociodemographic information. Third, reliance on self-reported symptoms introduces potential recall bias, a common challenge in population-based studies. Future research could mitigate this by integrating objective data sources such as electronic health records or wearable sensor data to enhance accuracy.

Fourth, our study considered only a subset of factors (i.e., education, age, and sex) as proxies for socioeconomic status, without accounting for additional confounders such as income, occupation (e.g., frontline vs. remote workers), political affiliation, or urbanization. These factors may influence both OS preference and health-related behaviors. Similarly, variations in mobile OS market penetration across different countries, as well as potential technical differences in app performance between iOS and Android, could also play a role in shaping symptom reporting patterns. Future research should incorporate these aspects using more detailed data collection approaches.

Fifth, the use of anonymous data precluded follow-up assessments, preventing confirmation or rejection of self-reported COVID-19 diagnoses. Future studies could implement opt-in longitudinal designs to enable verification of reported symptoms with objective clinical markers. Sixth, another limitation is that data was analyzed in an aggregated manner after the end of the COVID-19 pandemic. Different countries experienced multiple waves of the pandemic at varying intensities, and the observed country-specific associations might be influenced by differences in the timing and extent of these waves. In particular, in regions such as India and Bangladesh, COVID-19 prevalence estimates were affected by limited testing capacity, likely leading to an underestimation of the true infection burden in official case numbers. Future research should consider a longitudinal approach, evaluating trends in COVID-19 incidence and symptom reporting on an annual basis to account for dynamic epidemiological patterns.

Seventh, there was a brief two-month period (December 23, 2022–February 25, 2023) when data were only collected from Android users, not iOS users. This period coincided with the omicron variant surge in Europe. This omicron variant contained more mutations and seemed to avoid immunity more than previous variants. However, only 20 participants (12 from Europe) out of the total sample of 23,063 were included in this two-month period (see Fig. 3). This small number would not have introduced a meaningful bias to our results.

Finally, the study is based on cross-sectional data, which limits the ability to establish causal relationships between operating system choice and symptom-reporting behavior. While we observed significant differences between Android and iOS users, it remains unclear whether these differences are directly attributable to OS use or are confounded by underlying user characteristics. Future research employing mediation or moderation analyses, as well as longitudinal or experimental designs, would be valuable in further exploring these relationships.

In conclusion, our study compared self-reported COVID-19 symptoms and contact history between users of different mobile operating systems during the early period of the COVID-19 pandemic. Using data from over 23,000 individuals worldwide who used the *CoronaCheck* health app, we found that Android users were more likely to be classified as having a suspected COVID-19 infection compared to iOS users. While adjusting for sociodemographic factors explained part of these differences, many underlying mechanisms remain unclear, suggesting that additional factors such as differences in risk perception, health-seeking behaviors, and digital literacy may contribute to these disparities.

These findings highlight the importance of considering platform-specific differences when designing and interpreting mobile health (mHealth) studies. Mobile operating systems are more than just technological infrastructures; they reflect diverse user demographics, behaviors, and engagement patterns that can significantly impact health data collection. Tailoring digital health tools to accommodate these variations is crucial for improving user engagement, data accuracy, and public health interventions.

Further research is needed to better understand the factors driving differences between Android and iOS users, particularly in health reporting behavior. While our study provides valuable insights, several aspects require deeper investigation. One key limitation was self-selection bias, as participation was voluntary, and the sample may not fully represent all Android and iOS users. The overrepresentation of users from Germany, India, and South Africa (nearly 60%) limits generalizability, and the availability of *CoronaCheck* only in German and English may have contributed to a linguistically homogeneous user base, potentially influencing symptom reporting patterns. Future studies should aim for broader and more linguistically diverse recruitment strategies to improve representativeness.

Additionally, the cross-sectional nature of our study prevents causal inferences regarding OS-based differences in symptom reporting. Longitudinal research is needed to assess whether these differences persist over time and how evolving user behaviors influence health reporting. The integration of objective health data (e.g., electronic health records and wearable sensors) could help mitigate recall bias and improve accuracy. Moreover, moderation and mediation analyses (e.g., examining the interaction between OS and reported health outcomes) could provide deeper insights into the complex interplay of sociodemographic and technological factors.

Our study adjusted for key variables such as age, sex, education, and country, but other potential confounders—including income, occupation (e.g., frontline vs. remote workers), and urbanization—were not explicitly considered. These factors may influence both OS preference and health-related behaviors, and future studies should incorporate them into more detailed models. As mobile OS market penetration varies by country, additional research should explore regional differences in OS-based health engagement, particularly in areas with higher iOS adoption.

Another limitation was the lack of follow-up assessments due to the use of anonymous data, which prevented validation of self-reported COVID-19 diagnoses. Future studies should implement opt-in longitudinal designs that allow for symptom verification through clinical markers. Additionally, variations in COVID-19 waves across countries may have influenced symptom-reporting trends, necessitating a more dynamic epidemiological approach that evaluates trends annually.

Finally, as more empirical data become available, a directed acyclic graph (DAG) approach could systematically model complex interactions between sociodemographic, technological, and behavioral factors, helping to refine causal inferences^45^. Addressing these aspects will enhance the accuracy and inclusivity of mobile-based health surveillance, improving the reliability of digital epidemiology for both infectious diseases and chronic conditions.

## Methods

### Study design and setting

The development and set-up of the *CoronaCheck* app were described in detail previously^9^. For the present study, we conducted a cross-sectional analysis of self-reported questionnaire data collected by the app, which was offered globally during the COVID-19 pandemic through Google’s Play Store (Android) and Apple’s App Store (iOS). Given the urgency of the pandemic and the need for rapid insights into symptom reporting patterns across mobile platforms, a cross-sectional design was appropriate for capturing broad trends and identifying potential biases in digital health data collection.

To ensure broad usability and high response rates, the app assessed a wide range of COVID-19-associated symptoms using a standardized questionnaire that was intentionally kept concise. This approach minimized participant burden and maximized data completeness, as longer and more complex questionnaires risk lower engagement and increased dropout rates. The self-assessment tool, available in German and English, provided anonymous evaluations of potential COVID-19 infections based on regularly updated criteria from the German public health authority. Additionally, the app featured a news ticker on the COVID-19 pandemic and provided practical behavioral tips.

*CoronaCheck* was developed by a scientific collaboration of German universities and the Bavarian health authority. Analysis of the app data was approved by the ethics committee of the University of Würzburg, Germany (ethical approval No. 71/20-me). In general, the users of *CoronaCheck* read and approved the provided informed consent for app usage, and they had the option to provide further consent for their data to be used in scientific analyses. The university data protection officer was informed that only anonymous data would be collected and analyzed. The app was available in both Google’s Play and Apple’s App Store from April 4, 2020 to February 26, 2023.

**Fig. 1:**
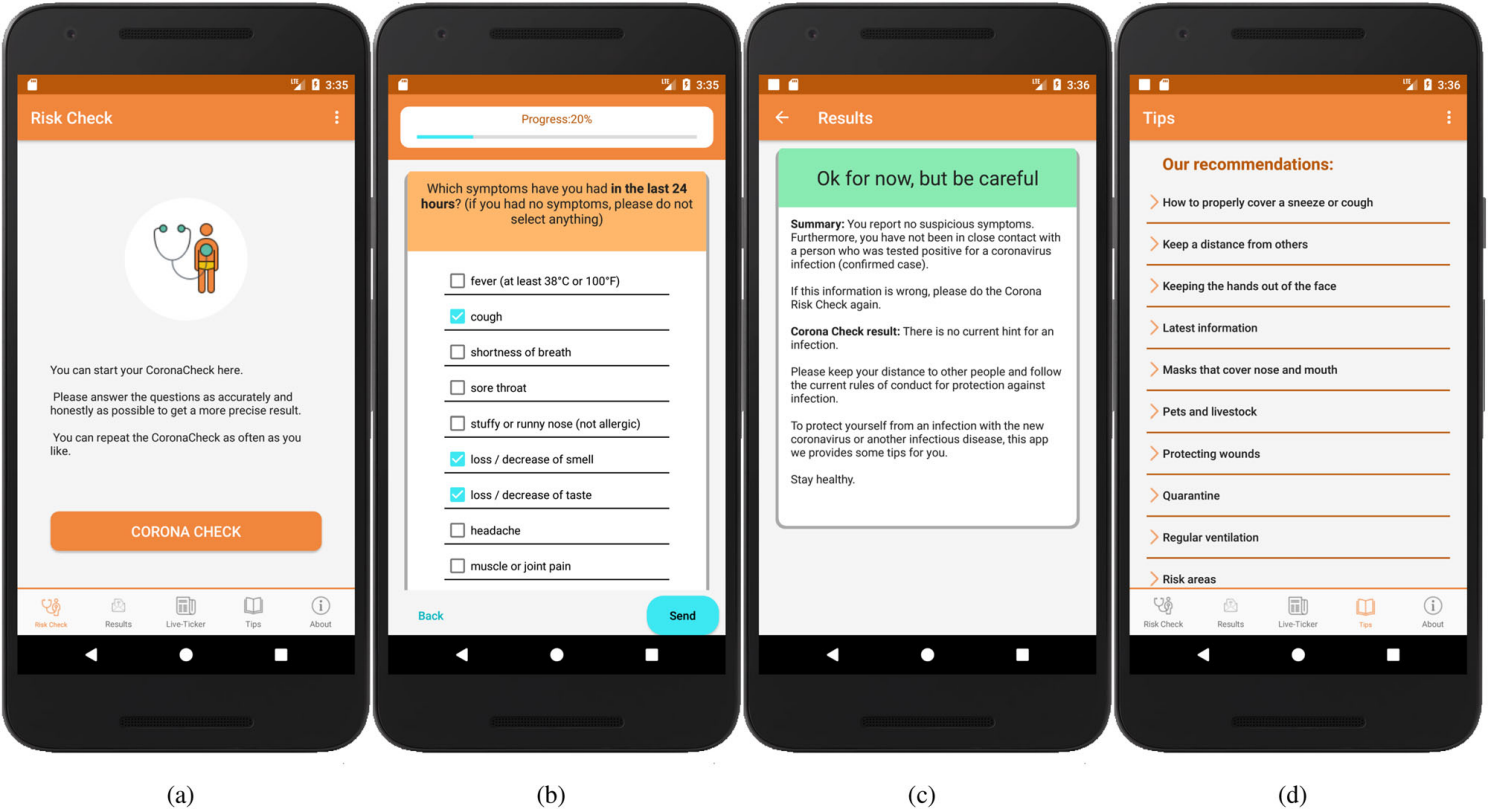
Screenshots of the Android *CoronaCheck* app illustrating its key functionalities. This figure presents screenshots of the Android *CoronaCheck* app illustrating its key functionalities across the user journey: **a** the cover screen, where users can initiate a COVID-19 self-assessment; **b** the screening process interface, where users report symptoms using a structured questionnaire; **c** the results screen that summarizes the user’s risk level and provides guidance on necessary actions; and (**d**) the tips section offering recommendations on COVID-19 prevention and management, including protective measures and guidance for infected individuals.

**Fig. 2:**
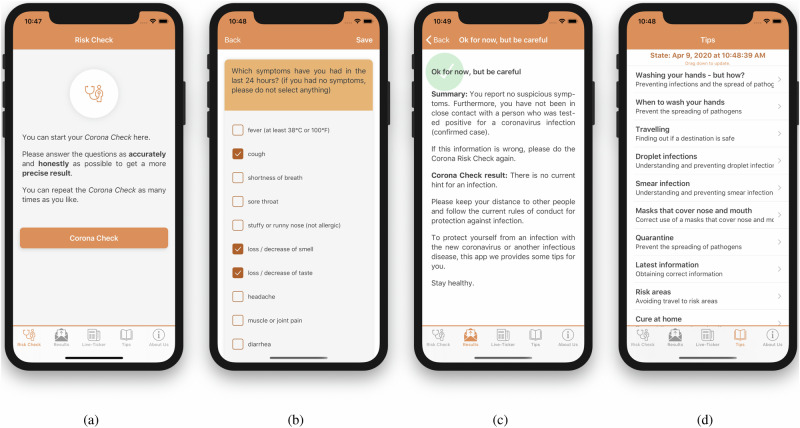
Screenshots of the iOS *CoronaCheck* app illustrating its key functionalities. This figure shows screenshots of the iOS *CoronaCheck* app illustrating its key functionalities across the user journey: **a** the cover screen, where users can initiate a COVID-19 self-assessment; **b** the screening process interface, where users report symptoms using a structured questionnaire; **c** the results screen that summarizes the user’s risk level and provides guidance on necessary actions; and **d** the tips section offering recommendations on COVID-19 prevention and management, including protective measures and guidance for infected individuals.

Figures 1 and 2a, b illustrate the key functionalities in Android and iOS of the *CoronaCheck* app. Figures 1 and 2a show the cover/title screen, where users can initiate a COVID-19 self-assessment. Figures 1 and 2b display the screening process, where users report symptoms in response to a structured questionnaire. Figures 1 and 2c present the results screen, summarizing the user’s risk level and providing guidance on necessary actions. Finally, Figs. 1 and 2d showcase the tips and recommendations section, which offers advice on COVID-19 prevention and management, including measures to reduce infection risk and recommendations for those infected.

Both the Android and iOS versions of the app were designed to maintain a consistent user interface and functionality. Only OS-specific requirements led to minimal differences in the UI, ensuring a uniform user experience across platforms.

### Study participants

Participants for this study were recruited among the users of the *CoronaCheck* app. All users worldwide, regardless of sociodemographic background, were invited to participate through an in-app consent process. The app ensured full anonymity in data collection, and users explicitly agreed that their anonymized data could be used for research purposes. Additionally, they had the option to provide further consent for their data to be included in scientific analyses. Recruitment was open from April 2020 to February 2023 and conducted entirely online through the app on Android or iOS. To maximize reach, the app was advertised through the institute’s official channels, including its website, Twitter, and Facebook. Participation was entirely voluntary, with no incentives provided.

### Assessment of suspected COVID-19 infection and related symptoms

The assessment of suspected COVID-19 infections was based on 11 symptoms related to COVID-19 (fever, sore throat, runny nose, cough, loss of smell, loss of taste, breathlessness, headache, muscle pain, diarrhea, and general weakness) and recent contacts to a person with a confirmed COVID-19 infection. After users completed the questionnaire, the app presented one of the following four conclusions about the potential COVID-19 infection status with recommendations for personal behavior and the protection of others^19^:

A. Suspected COVID-19 infection. This indicated that the user’s current symptoms and/or contact history suggested a high likelihood of COVID-19 infection.
B. Current symptoms without suspicion of COVID-19. The user had symptoms that may not necessarily indicate a COVID-19 infection.
C. Currently not symptomatic, but recent contact with a confirmed COVID-19 case. The currently asymptomatic user had recent contact with a person with a confirmed COVID-19 infection (by PCR), warranting caution and possibly self-quarantine.
D. No COVID-19 symptoms and no recent contact. “OK, for now, however, be careful.” The user reported no COVID-19-related symptoms and no suspicious COVID-19 contact history, however he/she should remain vigilant and adhere to preventive guidelines during the ongoing pandemic.

For users in Germany, contact information of the nearest local health authority was also offered.

### Assessment of sociodemographic variables

We assessed four key sociodemographic variables that are associated with differences in mortality, morbidity, risk and protective factors, and thus are crucial for population-based health research: sex, age, education level, and country^46^.

- Sex was categorized as follows: female, male, and diverse or not reported.
- Age was assessed in seven 10-year categories, ranging from 20 to 80+ years.
- Years in school were used as a proxy for the educational level to enable comparisons of international users from different educational systems. Years in school were categorized as less than 12 years, 12 or more years, or not reported.
- The country was derived from recorded GPS data, provided that participants had granted permission for its collection

### Data management

**Fig. 3:**
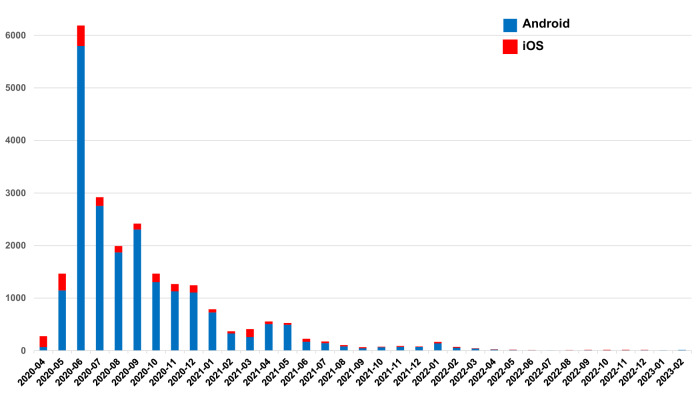
Response times by platform. This figure visualizes the temporal distribution of assessments from Android (blue) and iOS (red) users between April 2020 and February 2023. The figure shows that the majority of assessments were recorded within the first 12 months of the app’s availability.

The comparative analysis utilized app data covering the three-year period from the first entry on April 24, 2020 to the last entry on February 25, 2023. The first entry for iOS was on April 24, and Android on April 26, 2020. The last entry for iOS was on December 22, 2022 and Android on February 25, 2023. In total, 65,502 individuals had utilized the app, providing 89,901 completed questionnaires. Figure 3 shows the temporal distribution of assessments from Android (blue) and iOS (red) users in the available period from April 2020 to February 2023. While the majority of assessments were recorded within the first 12 months of the app’s availability, all available data from the entire period was included in the analysis to ensure a comprehensive evaluation. A user was able to complete the questionnaire also for others (e.g., relatives). Out of all, 41,027 users (58,734 completed questionnaires) gave consent for scientific use of the data. During data refinement, incomplete records (i.e., specifically those lacking responses in considered variables) were omitted to ensure consistency in user data. This resulted in 39,696 users (55,910 completed questionnaires): 36,404 used Android (51,496 completed questionnaires) and 3292 iOS (4414 completed questionnaires).

For the present analyses, we included the first assessment from each user ≥18 years of age, and only those that were for themselves, totaling 30,640 users (27,865 Android, 2775 iOS). Finally, data from users with ages <18 years were removed due to concerns about the accuracy of symptom reporting. It was unclear whether the symptoms were self-reported by the children or entered by their parents. As a result, assessments from a total of 23,063 users were evaluated: 20,753 Android, and 2310 iOS.

### Statistical analyses

In the descriptive analysis, we calculated absolute and relative frequencies (numbers and percentages). To examine the presence of potential differences in the number of symptoms, their presence, and COVID-19 detection results between Android and iOS users, the Mann–Whitney *U* test as well as Chi-squared tests were applied for categorical variables, and Student’s *t*-test for continuous variables.

To examine the association of the user platform on COVID-19-related outcomes, we conducted univariate and multiple logistic regression analyses for three different outcomes: (i) suspected COVID-19 infection, (ii) loss of smell (yes/no), and (iii) loss of taste (yes/no). The latter two symptoms are highly specific symptoms of COVID-19. We examined and adjusted the following variables as predictors: operating system (Android vs. iOS with iOS coded as 0 and Android coded as 1), age (with 20–29 coded as 0, 30–39 coded as 1, 40–49 coded as 2, 50–59 coded as 3, 60–69 coded as 4, 70–79 coded as 5, and 80+ coded as 6), schooling (with less than 12 years of schooling coded as 0, and 12 or more years coded as 1, and not reported coded as 2), sex (with male coded as 0, female coded as 1, and diverse and not reported coded as 2), and country of stay while using the app (with Germany as 0, India as 1, South Africa as 2, Netherlands as 3, Bangladesh as 4, aggregated other countries as 5, and not reported coded as 6).

We calculated both crude and adjusted odds ratios and corresponding 95% confidence intervals as a measure of statistical uncertainty. We followed an explorative (and not a confirmatory) statistical approach and did not determine a statistical significance level. Further, we did not adjust for multiple comparisons. All findings should be interpreted carefully.

## Supplementary information

Supplementary information

## Data Availability

The datasets used and/or analyzed during the current study are available from the corresponding author on reasonable request.

## Code availability

Inference statistics and data visualization were performed in SPSS (29.0.1) and Python (version 3.12.9). The code can be shared upon request to the corresponding author.
