# Supplementary material for: A comparison of self-reported COVID-19 symptoms between android and iOS CoronaCheck app users
Source: NPJ Digit Med. 2025 Apr 9;8:197. doi: 10.1038/s41746-025-01595-1 (PMC11982374; doi:10.1038/s41746-025-01595-1)
Supplement: Supplementary file 1 — Supplementary information [file 41746_2025_1595_MOESM1_ESM.pdf]

Supplementary Table 1: Distribution of Android and iOS users by country. This table presents the distribution of Android and iOS users across seven country groups included in our study. It provides the total number of participants for each group, along with the specific breakdown of Android and iOS users. The data highlights significant regional differences in smartphone usage, with Android being the dominant platform in most countries, except for the Netherlands, where iOS users constitute the majority.

Supplementary Table 1. Distribution of Android and iOS users by country

	**Android**	**iOS**
Germany (N = 7,358)	6512 (88.5%)	846 (11.5%)
India (N = 3,222)	3206 (99.5%)	16 (0.5%)
South Africa (N = 2,978)	2834 (95.2%)	144 (4.8%)
Netherlands (N = 477)	92 (19.3%)	385 (80.7%)
Bangladesh (N = 358)	329 (91.9%)	29 (8.1%)
Others (N = 2,372)	1939 (81.7%)	433 (18.3%)
Not reported (N = 6,298)	5841 (92.7%)	457 (7.3%)

Note: Total number of participants and the specific breakdown of Android and iOS users within each of the seven country groups included in our analysis.

Supplementary Table 2: Suspected COVID-19 infection risk comparison. The table illustrates the variation in suspected COVID-19 infection risk across different population groups. It compares the proportion of affected individuals within each category (e.g., mobile OS, sex, age group, education level, and country of residence). The "Risk difference" column represents the absolute percentage point difference between the two groups, providing insight into potential disparities in infection susceptibility.

Supplementary Table 2. Suspected COVID-19 infection risk comparison

**Factor 1: Risk of the outcome (n/N)**	**Factor 2: Risk for disease/symptom (n/N)**	**Risk difference**
Android; 4,987/20,753 = 24.0%	iOS; 257/2,310 = 11.1%	24.0% - 11.1% = 12.9%
Male; 3,656/14,668 = 24.9%	Female; 1,488/8,194 = 18.2%	24.9% - 18.2% = 6.7%
Male; 3,656/14,668 = 24.9%	Diverse and not reported; 100/201 = 49.8%	24.9% - 49.8% = -24.9%
Young (< 40y); 4,088/13,917 = 29.4%	Old (> 39y): 1,156/9,146 = 12.7%	29.4% - 12.7% = 16.7%
Education <12y; 1,442/7,364 = 19.6%	Education 12+; 2,261/9,961 = 22.7%	19.6% - 22.7% = -3.1%
Education <12y; 1,442/7,364 = 19.6%	Not reported; 1,841/6,008 = 30.6%	19.6% - 30.6% = 11%
Germany; 474/7,358 = 6.3%	Other countries; 4,770/15,705 = 30.4%	6.3% - 30.4% = -24.1%

Note: Comparative analysis of suspected COVID-19 infection risk across different factors. Each row contrasts two groups (e.g., Android vs. iOS users, male vs. female, younger vs. older individuals) in terms of the proportion of cases within each category. Risk Difference highlights the absolute percentage point difference between the groups.

Supplementary Table 3: Loss of smell risk comparison. This table shows the variation in loss of smell across different population groups. It compares the proportion of affected individuals within each category (e.g., mobile OS, sex, age group, education level, and country of residence). The "Risk difference" column represents the absolute percentage point difference between the two groups, providing insight into potential disparities in infection susceptibility.

Supplementary Table 3. Loss of smell risk comparison

Factor 1: Risk of the outcome (n/N)	Factor 2: Risk for disease/symptom (n/N)	Risk difference
Android; 2,655/20,753 = 12.8%	iOS; 141/2,310 = 6.1%	12.8% - 6.1% = 6.7%
Male; 1,923/14,668 = 13.1%	Female; 817/8,194 = 10.0%	13.1% - 10.0% = 3.1%
Male; 1,923/14,668 = 13.1%	Diverse and not reported; 56/201 = 27.9%	13.1% - 27.9% = -14.8%
Young (< 40y); 2,171/13,917 = 15.6%	Old (> 39y); 625/9,146 = 6.8%	15.6% - 6.8% = 8.8%
Education <12y; 785/7,364 = 10.7%	Education 12+; 1,321/9,961 = 13.7%	10.7% - 13.7% = -3%
Education <12y; 785/7,364 = 10.7%	Not reported; 955/6,008 = 15.9%	10.7% - 15.9% = -5.2%
Germany; 329/7,358 = 4.5%	Other countries; 2,467/15,705 = 15.7%	4.5% - 15.7% = 11.2%

Note: Comparative analysis of loss of smell risk across different factors. Each row contrasts two groups (e.g., Android vs. iOS users, male vs. female, younger vs. older individuals) in terms of the proportion of cases within each category. Risk Difference highlights the absolute percentage point difference between the groups.

Supplementary Table 4: Loss of taste risk comparison. the table shows the variation in loss of taste across different population groups. It compares the proportion of affected individuals within each category (e.g., mobile OS, sex, age group, education level, and country of residence). The "Risk difference" column represents the absolute percentage point difference between the two groups, providing insight into potential disparities in infection susceptibility.

Supplementary Table 4. Loss of taste risk comparison

Factor 1: Risk of the outcome (n/N)	Factor 2: Risk for disease/symptom (n/N)	Risk difference
Android; 2,586/20,753 = 12.5%	iOS; 139/2,310 = 6.0%	12.5% - 6.0% = 6.5%
Male; 1,812/14,668 = 12.4%	Female; 856/8,194 = 10.4%	12.4% - 10.4% = 2%
Male; 1,812/14,668 = 12.4%	Diverse and not reported; 58/201 = 28.4%	12.4% - 28.4% = -16%
Young (< 40y); 2,074/13,917 = 14.9%	Old (> 39y); 651/9,146 = 7.1%	14.9% - 7.1% = 7.8%
Education <12y; 767/7,364 = 10.4%	Education 12+; 1,267/9,961 = 13.1%	10.4% - 13.1% = -2.7%
Education <12y; 767/7,364 = 10.4%	Not reported; 967/6,008 = 16.1%	10.4% - 16.1% = -5.7%
Germany; 324/7,358 = 4.4%	Other countries; 2,401/15,705 = 15.3%	4.4% - 15.3% = -10.9%

Note: Comparative analysis of loss of taste risk across different factors. Each row contrasts two groups (e.g., Android vs. iOS users, male vs. female, younger vs. older individuals) in terms of the proportion of cases within each category. Risk Difference highlights the absolute percentage point difference between the groups.
